# Supplementary material for: Single Cell Transcriptomic and Chromatin Profiles Suggest Layer Vb Is the Only Layer With Shared Excitatory Cell Types in the Medial and Lateral Entorhinal Cortex
Source: Front Neural Circuits. 2022 Jan 26;15:806154. doi: 10.3389/fncir.2021.806154 (PMC8826650; doi:10.3389/fncir.2021.806154)
Supplement: Supplementary file 14 [file Data_Sheet_1.ZIP › Exc_vs_Inh_Motifs/homerResults/motif7.similar.html]

motif7

## Information for motif7

C
A
G
T
A
C
T
G
T
A
G
C
A
G
T
C
G
T
C
A
T
C
G
A
T
A
G
C
C
G
T
A
  
Reverse Opposite:  

C
G
A
T
A
T
C
G
A
G
C
T
C
A
G
T
A
C
T
G
A
T
C
G
G
T
A
C
G
T
C
A
  

|  |  |
| --- | --- |
| p-value: | 1e-310 |
| log p-value: | -7.150e+02 |
| Information Content per bp: | 1.809 |
| Number of Target Sequences with motif | 15805.0 |
| Percentage of Target Sequences with motif | 37.47% |
| Number of Background Sequences with motif | 3145.8 |
| Percentage of Background Sequences with motif | 28.94% |
| Average Position of motif in Targets | 320.7 +/- 243.4bp |
| Average Position of motif in Background | 295.9 +/- 233.1bp |
| Strand Bias (log2 ratio + to - strand density) | -0.0 |
| Multiplicity (# of sites on avg that occur together) | 1.32 |
| Motif File: | file (matrix) reverse opposite |

### Similar de novo motifs found

|  |  |  |  |  |  |  |  |
| --- | --- | --- | --- | --- | --- | --- | --- |
| Rank | Match Score | Redundant Motif | P-value | log P-value | % of Targets | % of Background | Motif file |
| 1 | 0.660 | C A G T G A C T G C A T A G T C A G T C C G A T C T A G T C A G T G A C G C T A C T G A T G A C | 1e-275 | -633.854159 | 34.99% | 27.10% | motif file (matrix) |
| 2 | 0.669 | A C G T C G T A A C T G A G T C C G T A C G T A A G T C C G T A | 1e-133 | -307.728031 | 44.19% | 38.32% | motif file (matrix) |
| 3 | 0.614 | A C G T A C G T A G T C A G T C C G T A C T A G A C T G C G T A | 1e-123 | -283.334344 | 9.47% | 6.46% | motif file (matrix) |
